# Identification, Expression and Activity Analyses of Five Novel Duck Beta-Defensins

**DOI:** 10.1371/journal.pone.0047743

**Published:** 2012-10-24

**Authors:** Deying Ma, Kexin Zhang, Mingyue Zhang, Shengnan Xin, Xiaoli Liu, Zongxi Han, Yuhao Shao, Shengwang Liu

**Affiliations:** 1 Colleage of Animal Science and Technology, Northeast Agricultural University, Harbin, PR China; 2 Division of Avian Infectious Diseases, State Key Laboratory of Veterinary Biotechnology, Harbin Veterinary Research Institute, Chinese Academy of Agricultural Sciences, Harbin, PR China; Saint Louis University, United States of America

## Abstract

In the current study, five novel avian β-defensins (AvBDs) were identified and characterized in tissues from Peking ducks (*Anas platyrhynchos*). The nucleotide sequences of these cDNAs comprised 198 bp, 182 bp, 201 bp, 204 bp, and 168 bp, and encoded 65, 60, 66, 67, and 55 amino acids, respectively. Homology, characterization and comparison of these genes with AvBD from other avian species confirmed that they were Apl_AvBD1, 3, 5, 6, and 16. Recombinant AvBDs were produced and purified by expressing these genes in *Escherichia coli*. In addition, peptides were synthesized according to the respective AvBD sequences. Investigation of the antibacterial activity of the Apl_AvBDs showed that all of them exhibited antibacterial activity against all 12 bacteria investigated (P<0.05 or P<0.01). In addition, the antibacterial activity of all of the AvBDs against *M. tetragenus* and *P. multocida* decreased significantly in the presence of 150 mM NaCl (P<0.01). None of the AvBDs showed hemolytic activity. Consistent with their broad-spectrum antibacterial activity, the five novel Apl_AvBDs inhibited replication of duck hepatitis virus (DHV) in vitro significantly (P<0.05). The mRNA expression of all five Apl_AvBD in most tissues, including immune organs and the liver, was upregulated in response to DHV infection at different time points. These findings provide evidence that these defensins activate the immune response to combat microbial infection.

## Introduction

Every year, many people throughout the world die as a result of microbial diseases. The consumption of poultry-derived products contaminated by bacteria or viruses is a major cause of food poisoning. Food safety has become a major concern for consumers, and this concern is reflected in the methods of food animal production and processing. Included in this new consumer advocacy is a call for a reduced use of antibiotics in preharvest pathogen control, because of concerns about the possible consumption of antibiotic residues and the emergence of antibiotic-resistant bacteria as a result of subtherapeutic use of antibiotics to control bacteria [1], [2]. As a consequence, other methods are needed to suppress microbial growth and infection in food animals. One of the alternatives may be to stimulate the innate immune system of the animal by dietary modulation, and therefore the identification and characterization of new innate immune effector molecules is required. Innate immunity in animals depends in large part on the activity of non-specific effector molecules. Among these, antimicrobial peptides (AMPs), which display activity against microbial pathogens, have been proposed as a novel control strategy to combat infections. The defensins are an important class of these peptides, and they have been identified and characterized in many species, including plants, invertebrates, and vertebrates [3]–[5]. The avian species express only β-defensins, which are named avian β-defensins (AvBDs). To date, more than 40 known isoforms of AvBDs have been identified in birds, including the chicken, duck, goose, quail, and other avian species [6]–[14]. All of these AvBDs have been shown to display a wide range of activity against bacteria and fungi [8]–[15]. In addition, most AvBDs can either be expressed constitutively or be induced in response to microbial infection, and their regulation is often dependent on the site of synthesis [12], [14], [15]. Although the expression of AvBDs in tissues has been reported, and its physiological significance has been suggested, the mechanisms by which this expression is regulated and the antimicrobial role mediated have not been established.

Among the avian species, ducks are infected frequently with viruses, including duck hepatitis virus (DHV), and they have been shown to shed viruses belonging to multiple subtypes [16]. To date, six AvBDs, named Apl-AvBD2, 4, 7, 9, 10, and 12, have been identified from ducks, and antiviral activity against DHV has been found in Apl-AvBD4, 7, and 12 [9], [10], [12]. Enhancement of the expression of apl-AvBD4, 7, and 12 by DHV in several tissues of ducks was found in our previous study [12]. In the present study, another five novel Apl_AvBDs from ducks (*Anas platyrhynchos*) have been isolated and characterized. Furthermore, we examined whether expression of these five novel Apl_AvBDs were changed in response to DHV infection.

## Materials and Methods

### Ethics Statement

All animal experimental procedures were approved by the Ethical and Animal Welfare Committee of Heilongjiang province, China.

### Animals

Five 21-day-old specific pathogen free (SPF) Peking ducklings and thirty 11-day-old SPF Peking ducklings were obtained from the Laboratory Animal Center, Harbin Veterinary Research Institute, the Chinese Academy of Agricultural Sciences, China. The birds were maintained in isolators with negative pressure, and food and water were provided ad libitum.

### RNA Extraction, Reverse Transcriptase Polymerase Chain Reaction Amplification, and Sequencing

Approximately 1 g of each of 23 tissues, namely the skin, tongue, esophagus, larynx, glandular stomach, muscular stomach, trachea, lung, heart, liver, kidney, breast muscle, spleen, bone marrow, bursa of Fabricius, Harderian glands, thymus, cecal tonsil, small intestine, cecum, rectum, large intestine, and pancreas, was obtained from five healthy 21-day-old SPF ducklings. The samples were processed to yield tissue fluid, and the total cellular RNAs were extracted from 100 µL aliquots of the respective tissue fluid using TRIzol reagent (Invitrogen, Beijing, PR China) according to the manufacturer’s instructions. Reverse transcriptions (RT) were performed using oligo-dT primers in a 40 µL reaction mixture containing 20 µL RNA. The specific cDNAs obtained were amplified by PCR using Ex-Taq polymerase (TAKARA Bio Inc., Otsu, Shiga, Japan) with five sets of primers designed internally on the basis of the coding sequences of chicken AvBD1, 3, 5, 6 (Table 1), respectively. In addition, cDNAs obtained from bone marrow were amplified by PCR with a set of primers for duck β-actin (Table 1). The PCR protocol was as follows: an initial denaturation for 5 min at 95°C followed by 30 cycles of denaturation at 94°C for 30 s, annealing at 50°C for 30 s, and polymerization at 72°C for 1 min. The final polymerization step was performed at 72°C for 10 min. The PCR products were cloned into the pMD18-T vector (TAKARA) to confirm amplification, followed by sequencing of the recombinant plasmids.

**Table 1:** PCR primer sequences and predicted product lengths.

Target mRNA	Sense primer (5′-3′)	Antisense primer (5′-3′)	Product size (bp)	GenBank accession no
Apl_ AvBD1 (RT-PCR)	AAACCATGCGGATCGTGTACCTGC	ATGGGGGTTGTTTCCAGGAGC	264	JQ359441
Apl_ AvBD3 (RT-PCR)	GAACTGCCACTCAGTGCAGAAT	ATGGGGGTTGTTTCCAGGAGC	183	-
Apl_ AvBD5 (RT-PCR)	ATGCAGATCCTGCCTCTCCTCTTTGCT	TCAGGAATACCATCGGCTCCGGCAGCAGAA	201	JF949720
Apl_ AvBD6 (RT-PCR)	ATGAGGATCCTTTACCTG	TCAGGCCCACCTGTTCCT	204	JQ359446
Apl_ AvBD16 (RT-PCR)	CCTTCTTCCTCTTGTTTCTCCAG	ATGGGGGTTGTTTCCAGGAGC	216	JQ359445
Duck β-actin (RT-PCR and real time PCR)	CCGTAAGGACCTGTACGCCAACAC	GCTGATCCACATCTGCTGGAAGG	208	AY251275
Apl_ AvBD1 (real time PCR)	GAAACAAGGAGAAATGTCATCG	ATGGGGGTTGTTTCCAGGAGC	183	JQ359441
Apl_ AvBD3 (real time PCR)	GAACTGCCACTCAGTGCAGAAT	ATGGGGGTTGTTTCCAGGAGC	183	–
Anser AvBD5 (real time PCR)	GCTGTCCCTTGCTCGAGGATT	GGAATACCATCGGCTCCGGC	139	JF949720
Apl_ AvBD6 (real time PCR)	GTCAGCCCTACTTTTCCAGC	GCCCACCTGTTCCTCACAC	143	JQ359446
Apl_ AvBD16 (real time PCR)	GAAACTCTGTCCTCTGCAGAAT	ATGGGGGTTGTTTCCAGGAGC	183	JQ359445
Apl_ AvBD1 (protein expression)	GAATTC ATGCGGATCGTGTACCT	GTCGAC TCAACCAAATATC	210	JQ359441
Apl_ AvBD3(protein expression)	GGATCC ATGACTGCCACTCAGTG	GTCGAC TCAATGGGGGTTGTTTC	198	–
Apl_ AvBD5 (protein expression)	GAATTC ATGCAGATCCTGCCTCTC	GTCGAC TCAGGAATACCATCGGCTCCGGCA	208	JF949720
Apl_ AvBD 6 (protein expression)	GAATTC ATGAGGATCCTTTACCTG	GTCGAC TCAGGCCCACCTGTTCCT	216	JQ359446
Apl_ AvBD16 (protein expression)	GGATCC ATGTTCTTCCTCTTGTTTCT	GTCGAC TCAACCCCATGTCCT	168	JQ359445

### Sequence Analysis of Apl_AvBD cDNAs

Basic searches were conducted with a local alignment search tool (BLAST) using the five entire AvBD cDNAs obtained from the ducks. Sequences of the other known AvBDs were selected for comparison with the five novel Apl_AvBDs. The signal peptides of the five novel Apl_AvBDs were analyzed using the SignalP 3.0 server (http://www.cbs.dtu.dk/Services/signalP).

### Protein Expression and Purification

The cDNA fragments that encode the Apl_AvBDs were amplified by PCR from the plasmids described above, using the primers for protein expression shown in Table 1. The PCR products, which contained the coding sequence of either Apl_AvBD1, 5, or 6 flanked by *Eco*R I/*Sal* I, and either Apl_AvBD3 or 16 flanked by *Bam*H I/*Sal* I, were inserted into the pGEX-6p-1 vector (Amersham) at the respective sites. The resultant plasmids were designated recombinant Apl_AvBD1, 3, 5, 6, or 16-pGEX, respectively, and sequenced again. The constructs that were confirmed to contain these Apl_AvBDs were transformed into competent *Escherichia coli* BL21 (DE3) cells. Expression of the fusion proteins was induced with isopropyl β-D-1-thiogalactopyranoside (IPTG), and the proteins were purified using a purification and refolding kit (no. 70123-3; Novagen, Darmstadt, Germany), according to the manufacturer’s instructions. Briefly, the induced culture was harvested by centrifugation at 6500 *g* for 15 min at 4°C, and the supernatant was removed and discarded. Then, the cell pellet was weighted and resuspended in 1× Inclusion Body (IB) Wash Buffer (20 mM Tris-HCl pH 7.5, 10 mM EDTA, 1% Triton X-100) and sonicated after a 15-min incubation at 30°C with lysozyme. The inclusion bodies were collected and weighted by centrifugation at 10,000 *g* for 10 min. Then, they were resuspended in 1×IB solubilization buffer supplemented with 0.3% N-lauroylsarcosine. The supernatant containing the fusion proteins were filtered through a cellulose acetate filtration membrane with a pore size of 0.45 µm and then passed through an affinity chromatography column of glutathione Sepharose 4B (Amersham) equilibrated with PBST (PBS +1% Triton 100). The column was washed with 6 bed volumes of PBS to remove contaminating proteins. The recombinant fusion proteins were then eluted with 10 ml of 50 mM of Tris-HCl buffer containing 10 mM reduced glutathione, pH 8.0. The fusion proteins were concentrated using Centricon Microconcentrators (Millipore, Beijing, China) with a molecular weight cutoff of 10 kDa. The fusion proteins were resolved by 12% sodium dodecyl sulfate–polyacrylamide gel electrophoresis (SDS-PAGE) at 80 V, using the Mini-protean III system (Bio-Rad, Beijing, China) and stained with Coomassie brilliant blue R-250 [17]. The concentration of the purified proteins was determined by the Bradford method using bovine serum albumin as the standard [18].

### Peptide Synthesis

The linear N-terminal-acetylated forms of the predicted mature Apl_AvBD1, 3, 5, 6, and 16 peptides were custom synthesized and purified using high performance liquid chromatography (HPLC**)** at GL Biochem Ltd (Shanghai, China) (http://www.glschina.com/en/profile).

### Antibacterial Activity

Colony counting assays were performed according to the methods used in previous studies [11] to investigate the antimicrobial activities of glutathione-S-transferase (GST) and the recombinant AvBDs (rAvBDs), and synthetic AvBDs (sAvBDs) from ducks against four strains of Gram-positive bacteria: *Micrococcus tetragenus* (ATCC2835), *Lactobacillus* (ATCC33222), *Staphylococcus aureus* (ATCC 29213), and *Bacillus cereus* (ATCC 9193); and eight strains of Gram-negative bacteria: *Bordetella bronchiseptica* (S80103), *Proteus mirabilis* (ATCC29245), *Pseudomonas aeruginosa* (ATCC 9027), *Pasteurella multocida* (ATCC 6529), *E. coli* BL21 (DE3), *Salmonella pullorum* (C79-11-S11), *Salmonella choleraesuis* (CVCC 2140), and *Salmonella enteritidis* (ATCC 3021). All of the bacterial strains used were obtained from Chinese Institute of Veterinary Drug Control. The antimicrobial activities of both rAvBD and sAvBD were determined by colony counting assay. Briefly, a 10 µL of diluted bacterial culture (diluted 1000-fold in the appropriate medium) was treated with 250 µL of rAvBD or sAvBD (0 to 100 µg/mL in PBS) in polypropylene microtiter plates and preincubated for 3 h at conditions suited to the investigated strain; 100 µL of minimal medium was added after 3 h and the plates were incubated for 12–18 h further. Then, the sample was rediluted 100-to-10^6^-fold in minimal medium, transferred to TSA plates and colonies were counted after 24 h of incubation. Bactericidal activity was calculated as a percentage of CFU of bacteria not exposed to antimicrobial peptides but subjected to the same experimental conditions. Results from the antimicrobial assay represent the mean of the three independent experiments, with three replicates per experiment.

### Effect of Ionic Strength on the Antibacterial Activity

The effect of ionic strength on the antibacterial activity against *M. tetragenus* and *P. multocida* of the peptides at concentration of either of 25 or 100 µg/mL was studied. A 250 µL of peptides were incubated separately with 10 µL of diluted either *S. aureus or P. mirabilis* for 3 h at conditions suited to the investigated strain, with different concentrations of NaCl (0, 50, 100, 150 mM) in 10 mM sodium phosphate buffer, pH 7.4. Bactericidal activity was calculated as described above. Results from the antimicrobial assay represent the mean of three independent experiments, with three replicates per experiment.

### Antiviral Activity against DHV-1 in vitro

The antiviral activity of rApl_AvBD1, 3, 5, 6, and 16 against DHV-1 strain Du/CH/LBJ/090809 (data not shown) was determined according to methods described for neutralization assays [19]. Briefly, the DHV-1 (10^8^ median embryo infectious doses, EID_50_) was serially diluted 10-fold and treated with equal volumes of GST or rAvBDs (300 µg/mL in PBS) at 37°C for 60 min. The mixture was inoculated into 11-day-old specific pathogen free (SPF) duck embryos, with 200 µL administered per duck. The inoculated duck embryos were incubated at 37°C and observed for 7 days. Mortality was recorded every 8 h. The eggs that died between 24 h and 7 days post inoculation, or those that showed characteristic embryonic changes, such as swelling, stunting, or hemorrhage, were recorded as positive for DHV-1 infection. All antiviral activity assays were performed in three independent experiments, with five replicates per experiment.

### Hemolysis Assay

The hemolytic activities of the defensins were determined essentially as described previously [20]–[22]. Briefly, fresh duck blood was collected, washed twice with PBS, and diluted to 0.5% in PBS with and without the addition of 10% PBS, followed by dispensing 90 µL into 96-well plates. Different concentrations of peptides (10 µL) dissolved in 0.01% acetic acid were added in duplicate to the cells and incubated at 37°C for 2 h. Following centrifugation at 800×*g* for 10 min, the supernatants were transferred to new 96-well plates and monitored for released hemoglobin by measuring the absorbance at 405 nm. Controls for 0 and 100% hemolysis consisted of cells suspended in PBS only and in 1% Triton X-100, respectively. All hemolysis assays were performed in three independent experiments, with three replicates per experiment. The percentage of hemolysis was calculated as: [(*A*_405 nm, peptide_ – *A*_405 nm, PBS_)/(*A*_405 nm, 1% Triton X-100_– *A*_405 nm, PBS_)] × 100%.

### Viral Challenge and Expression Analysis in Ducks

The remaining thirty 11-day-old healthy ducklings mentioned above were used to study the tissue distribution of the AvBD mRNAs in healthy ducklings and in ducklings responding to challenge with DHV. The ducklings were challenged by intramuscular inoculation with DHV-1 (10^8^ EID_50_ of Du/CH/LBJ/090809). The viral challenge of the experimental animals, conducted in vivo, was in concordance with the accepted welfare guidelines of the institution. Five birds were sacrificed at 0 h (these served as a control), and at 24, 32, 48, 72, and 96 h after infection, respectively. The bursa of Fabricius, spleen, thymus, cecal tonsil, bone marrow, liver, and kidneys of each bird were collected, rinsed in cold sterile saline, snap-frozen in liquid nitrogen, and stored at −70°C until further use.

In order to confirm the infection of the ducklings, DHV-1 was isolated and RT-PCR was performed by targeting the VP0 gene of DHV-1 in the liver of the infected ducklings, followed by sequencing analysis (data not shown).

### Real-time RT-PCR

The levels of the 5 Apl_AvBD, and β-actin mRNA in tissues from the ducklings were measured with the real-time RT-PCR method using SYBR Premix EX Taq™ (Takara Biotechnology, Dalian, China). Equal amounts of tissue (1 g) were used to produce the tissue fluid. The total cellular RNAs were extracted and RT was performed as described above. Real-time PCR was performed with the LightCycler® 480 II Real-Time PCR system (Roche, Switzerland). Serial 10-fold dilutions of a standard plasmid that contained either Apl_AvBD1, 3, 5, 6, 16, or β-actin were prepared in order to produce a standard curve from 10^3^ to 10^10^ copies per µL for each target cDNA, as described by Sadeyen et al. [23]. The real-time PCR reactions (25 µL) contained 1 µL (2.5 µmol) of each primer, 12.5 µL of 2×SYBRII Green, 8.5 µL nuclease-free water, and 2 µL either template cDNA sample or a known concentration of standard plasmid. The primers are shown in Table 1. The reaction was performed at 95°C for 30 s, followed by 40 cycles of 95°C for 5 s and 60°C for 30 s. The PCR amplification was followed by melting curve analysis, in which the temperature was decreased from 95 to 65°C at a rate of 0.2°C/sec, with continuous monitoring of the decline in fluorescence. All cDNA amplifications were performed in triplicate. Only PCR products that showed the unique melting temperature, which confirmed a unique PCR product, were retained for further automated quantitative analysis. The results were expressed finally for each sample as the copy number of each target cDNA normalized to 10^4^ times the copy number of the housekeeping cDNA β-actin, using the following formula: (target cDNA copy number/β-actin cDNA copy number)×10^4^. The specificity of the reaction was checked by cloning and sequencing three independent PCR products along with the melting curve analysis. All assays were performed three times, with three replicates per experiment.

### Statistics

Data are expressed as the mean±SD. The statistical analyses, where appropriate, were performed using one-way analysis of variance (ANOVA) using the GLM procedure of SAS software [24]. A difference with P<0.05 was considered to be statistically significant.

The nucleotide sequences of Apl_AvBD1, 5, 6, and 16 obtained in this study are available from GenBank under the accession numbers: JQ359441, JF949720, JQ359446, and JQ359445, respectively.

## Results

### Identification and Characterization of Five Novel Duck β-defensin Genes

**Figure 1:**
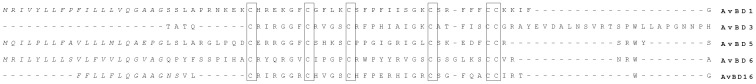
Deduced amino acid sequence alignment of the five novel avian β-defensins (AvBDs) from the duck. Signal sequences of the Apl_AvBDs are italic. The conserved six cysteines (C) are framed. *Dashes* indicate no identical or conserved residues observed.

Twenty-three tissues were used to identify potential defensins in the duck, using five sets of primers (Table 1), designed internally on the basis of the coding sequences of chicken AvBD1, 3, 5, 6. Since duck AvBD2, 4, 7, 9, 10, and 12 have been identified in our previous study [9], [10], [12]. Five novel Apl AvBDs were identified, in both bone marrow and lung, using these five sets of primers. The complete nucleotide sequences of the first, third, and fourth cDNAs contained open reading frames (ORFs) of 198 bp, 201 bp, and 204 bp, and encoded 65, 66, and 67 amino acids, respectively (Fig. 1). The cDNA fragments of the second and the last cDNAs contained 182 bp and 168 bp, and encoded 60 and 55 amino acids, respectively (Fig. 1). The predicted amino acid sequences of all of these peptides contained the six cysteine residues and the GXC motif that are conserved across all β-defensins (Fig. 1). The full-length sequence of the first peptide (65 amino acids) showed the highest percentage of amino acid homology (78.0%) with AvBD1 from the ostrich, and also showed a high percentage of amino acid homology (67.7%) with AvBD1 from the chicken. The full-length sequences of the second and the fourth peptides shared 100% amino acid homology with chicken AvBD3 and chicken AvBD6, respectively. The full-length sequence of the third peptide (66 amino acids) showed the highest percentage of amino acid homology (97.0%) with chicken AvBD5, and showed a high percentage of amino acid homology (87.9%) with AvBD5 from the goose. The full-length sequence of the last peptide (55 amino acids) showed the highest percentage of amino acid homology (62.2%) with chicken AvBD3, and low amino acid homology with the other AvBDs. On the basis of these analyses, the five novel cDNAs were identified conclusively as β-defensin orthologs in the duck, and were named Apl_AvBD1, Apl_AvBD3, Apl_AvBD5, Apl_AvBD6, and Apl_AvBD16, respectively. Furthermore, other two sets of primers, designed internally on the basis of the coding sequences of chicken AvBD3 were used to amplify full-length sequences of both AvBD3 and 16 in the study. Unfortunetly, neither of them were identified (data not shown).

### Expression and Purification of Recombinant Apl_AvBDs

**Figure 2:**
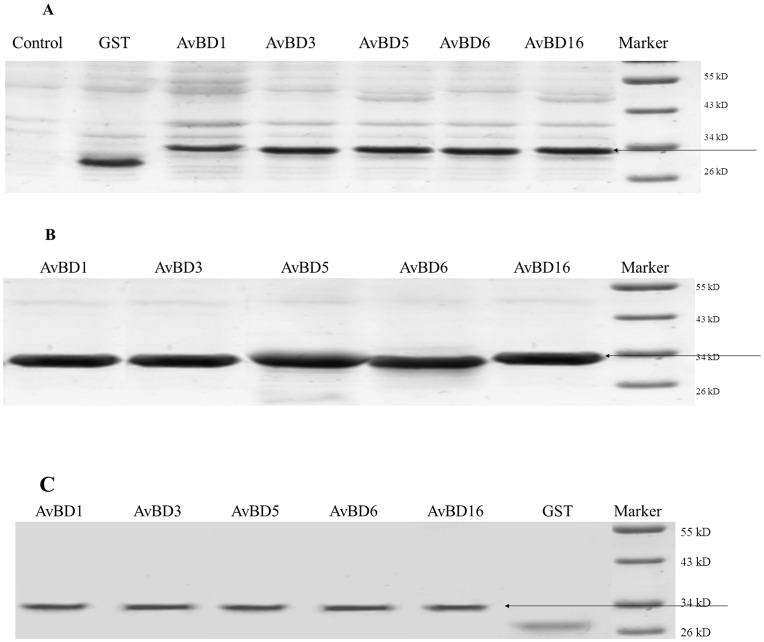
SDS–PAGE analysis of glutathione-S-transferase (GST)-tagged recombinant Apl_avian β-defensin (Apl_AvBD) proteins expressed in *E. coli* BL21 (DE3) cells. (A) total protein from BL21 containing Apl_AvBD1, 3, 5, 6, 16, and GST with IPTG induction, respectively; (B) inclusion bodies with Apl_AvBD1, 3, 5, 6, 16, and GST respectively; (C) purified proteins of Apl_AvBD1, 3, 5, 6, 16, and GST with IPTG induction, respectively. IPTG, isopropyl-beta-D-thiogalactoside.

High levels of expression of all five Apl_AvBDs were noted in *E. coli* after induction with 0.6 mM IPTG (Fig. 2a). All of the recombinant AvBDs-pGEX (molecular weight, 32 [kD]) were produced as inclusion bodies. These peptides were purified and visualized as a pronounced band on SDS-PAGE gels (Fig. 2b).

### Antibacterial Properties of Apl_AvBDs

**Figure 3:**
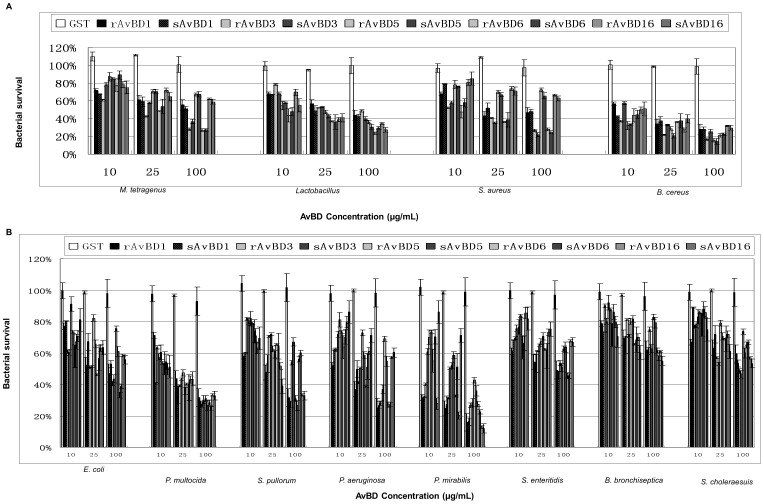
Antimicrobial activity of glutathione-S-transferase (GST), recombinant Apl_avian β-defensins (r Apl_AvBDs), and respective synthetic Apl_AvBDs (sApl_AvBDs) against bacteria. (A) Gram-positive bacteria; (B) Gram-negative bacteria. Bactericidal activity was calculated as the percentage of colony counts of bacteria not exposed to antimicrobial peptides but subjected to the same experimental conditions. All studies were performed in three independent experiments with three replicates per experiment, and each bar represents the mean ± SD. The data were analyzed using SAS [24].

To investigate the antibacterial properties of the novel Apl_AvBDs, the rApl_AvBDs (1, 3, 5, 6, and 16) were produced and purified. In addition, putative mature Apl_AvBDs (1, 3, 5, 6, and 16) were synthesized commercially and purified to >95% purity. The dose-dependent survival of bacteria treated with GST, rApl_AvBD, and sApl_AvBD peptides was determined by colony counting assays at concentrations of 10–100 µg/mL. The data are depicted in Fig. 3 and show that GST had no bactericidal activity against any of the bacterial strains investigated. In contrast, all of the Apl_AvBDs showed dose-dependent bactericidal activity against all of the bacterial strains investigated (P<0.05 or P<0.01). In addition, each rApl_AvBD peptide showed similar bactericidal activity to the respective sApl_AvBD peptide (P>0.05). High and similar antibacterial activities were found for all the Apl_AvBDs against *B. cereus*, *P. mirabilis* and *P. multocida*, and the survival rate of all three bacteria was lower than 40% at concentration of 100 µg/mL (P<0.01). Furthermore, both Apl_AvBD3 and 6 showed high antibacterial activity against *S. aureus* and *M. tetragenus*, and Apl_AvBD1, 3 and 6 showed high antibacterial activity against *P. aeruginosa* when compared with GST (P<0.05 or P<0.01). However, low but significant bactericidal activities were found for all of these Apl_AvBDs against *S. choleraesuis* and *B. bronchiseptica* (P<0.05).

### Effect of Ionic Strength on Antibacterial Activity of Apl_AvBDs

**Figure 4:**
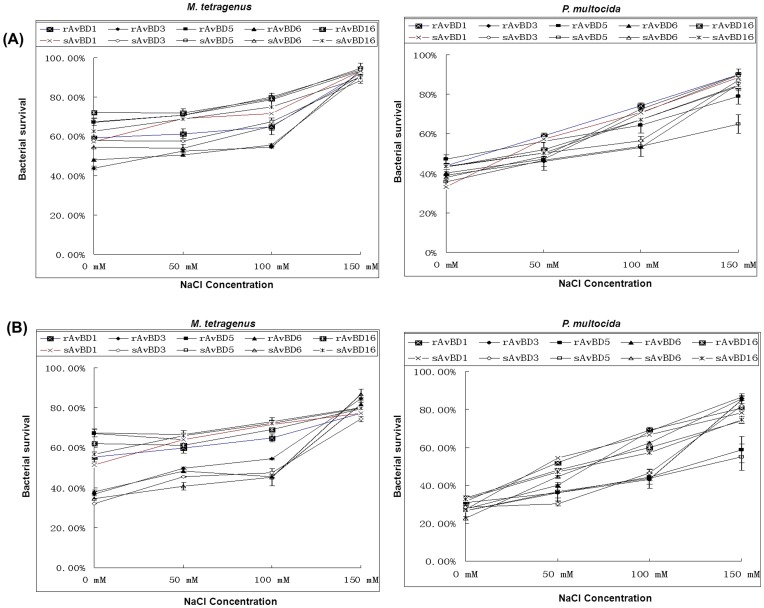
Effects of salinity on the antibacterial activity of Apl_avian β-defensins (Apl_AvBDs) against *M. tetragenus* or *P. multocida*. (A) 25 µg/mL Apl_AvBDs; (B) 100 µg/mL Apl_AvBDs. Apl_AvBDs were incubated separately with either *M. tetragenus* or *P. multocida* for 3 h in the presence of 0, 50, 100, and 150 mM NaCl. All assays were performed in three independent experiments with three replicates per experiment, and each bar represents the mean ± SD. The data were analyzed using SAS [24].

The antibacterial activity against *M. tetragenus* and *P. multocida* of the Apl_AvBDs was determined in the presence of 0, 50, 100, and 150 mM NaCl at concentrations of 25 and 100 µg/mL, respectivrly. It was shown that all of the Apl_AvBDs (25 and 100 µg/mL) retained similar bactericidal activity against *M. tetragenus* at NaCl concentrations that ranged from 0 to 100 mM. However, the antibacterial activity against *M. tetragenus* of these Apl_AvBDs decreased significantly in the presence of 150 mM NaCl (P<0.05). Furthermore, the antibacterial activity against *P. multocida* of all of the Apl_AvBDs at either concentration was dependent on the NaCl concentration, and at 150 mM NaCl the antibacterial activity against *P. multocida* decreased significantly (P<0.01) (Fig. 4).

### Antiviral Activities of Apl_AvBDs against DHV-1 in vitro

**Figure 5:**
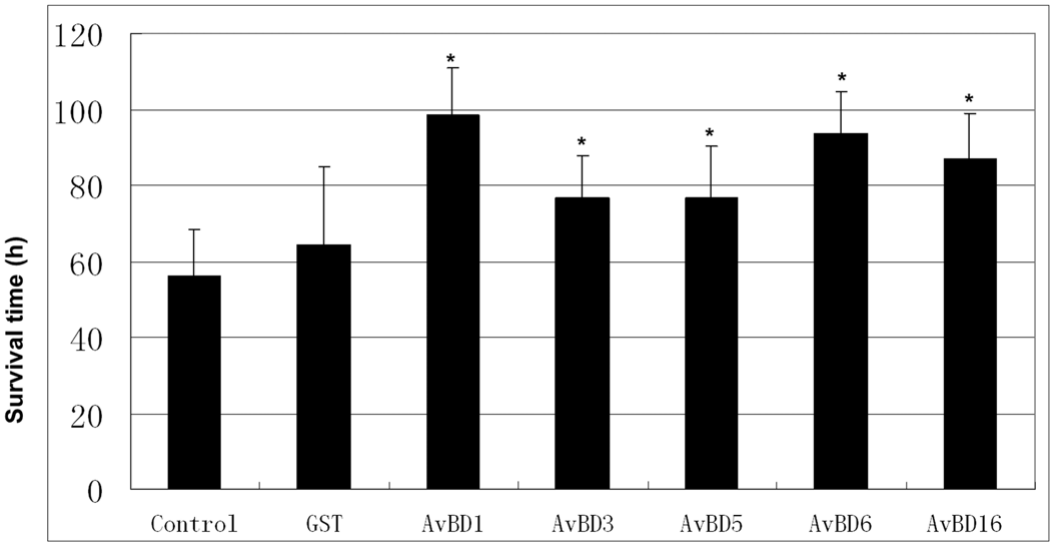
Antiviral activities of glutathione-S-transferase (GST), and recombinant Apl_avian β-defensins (rApl_AvBDs) against duck hepatitis virus. All assays were performed in three independent experiments, with five replicates per experiment, and each bar represents the mean ± SD. The data were analyzed using SAS [24], and P<0.05 is indicated by an asterisk “*”.

The dose-dependent survival time of duck embryos inoculated with GST or rApl_AvBD-treated DHV was investigated in the present study (Fig. 5). DHV-1 was serially diluted and treated with equal volumes of GST or rAvBDs (300 µg/mL in PBS). Mortality was recorded every 8 h. It was shown that GST had little effect on the survival time of duck embryos, when compared with the control (P>0.05). In contrast, the survival time of duck embryos was prolonged significantly by all of the rApl_AvBDs (P<0.05), when compared with both the control and GST.

### Cytotoxocity of Apl_AvBDs

**Figure 6:**
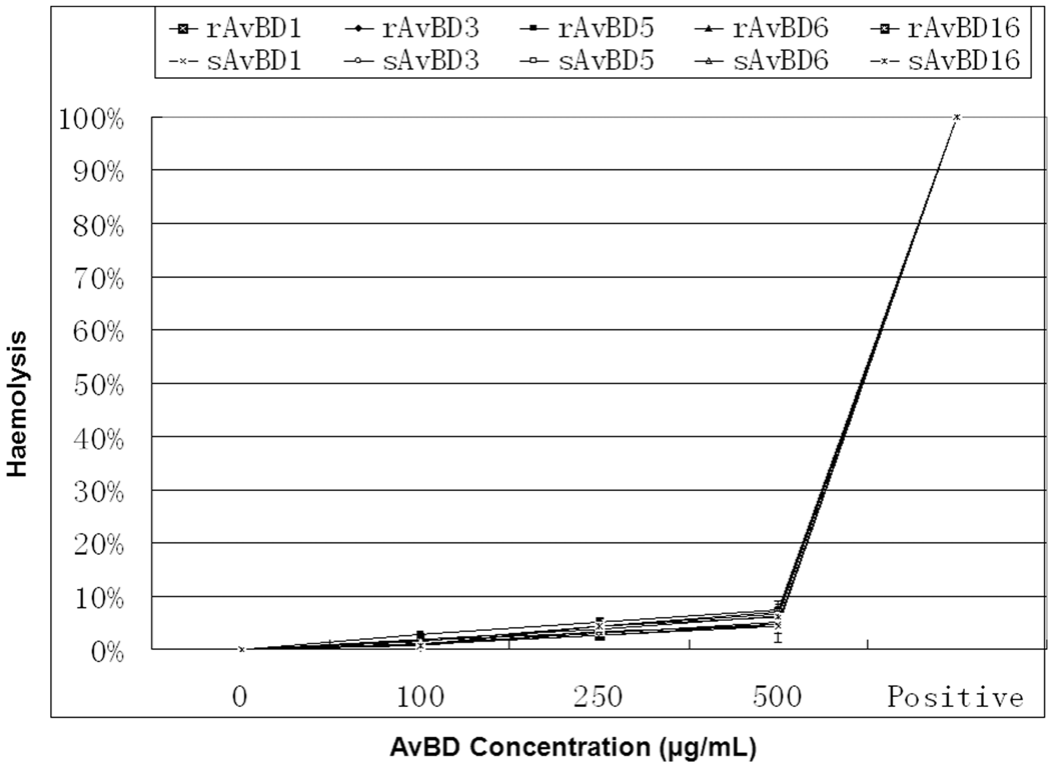
Hemolytic activities of duck avian β-defensins (AvBDs). Freshly isolated duck red blood cells were incubated with different concentrations of AvBDs (0–500 µg/mL). Release of hemoglobin, as a measure of hemolysis, was measured at 405 nm. Release of hemoglobin upon addition of 1% Triton X-100 was set at 100%. The percentage of hemolysis was calculated as [(*A*_405 nm, peptide_ – *A*_405 nm, PBS_)/(*A*_405 nm, 1% Triton X-100_– *A*_405 nm, PBS_)] × 100%. All assays were performed in three independent experiments, with three replicates per experiment, and each point is the mean ± SD. The data were analyzed using SAS [24].

Freshly isolated duck erythrocytes were collected and incubated with rApl_AvBDs or sApl_AvBDs, together with Triton X-100 as a positive reference. The release of hemoglobin was measured as an indication of the hemolytic activity of these peptides. As shown in Fig. 6, little hemolysis of duck erythrocytes was observed at any concentration of any of the Apl_AvBDs, when compared with the negative control (P>0.05).

### Effects of DHV Infection on Expression of Defensin mRNA

**Figure 7:**
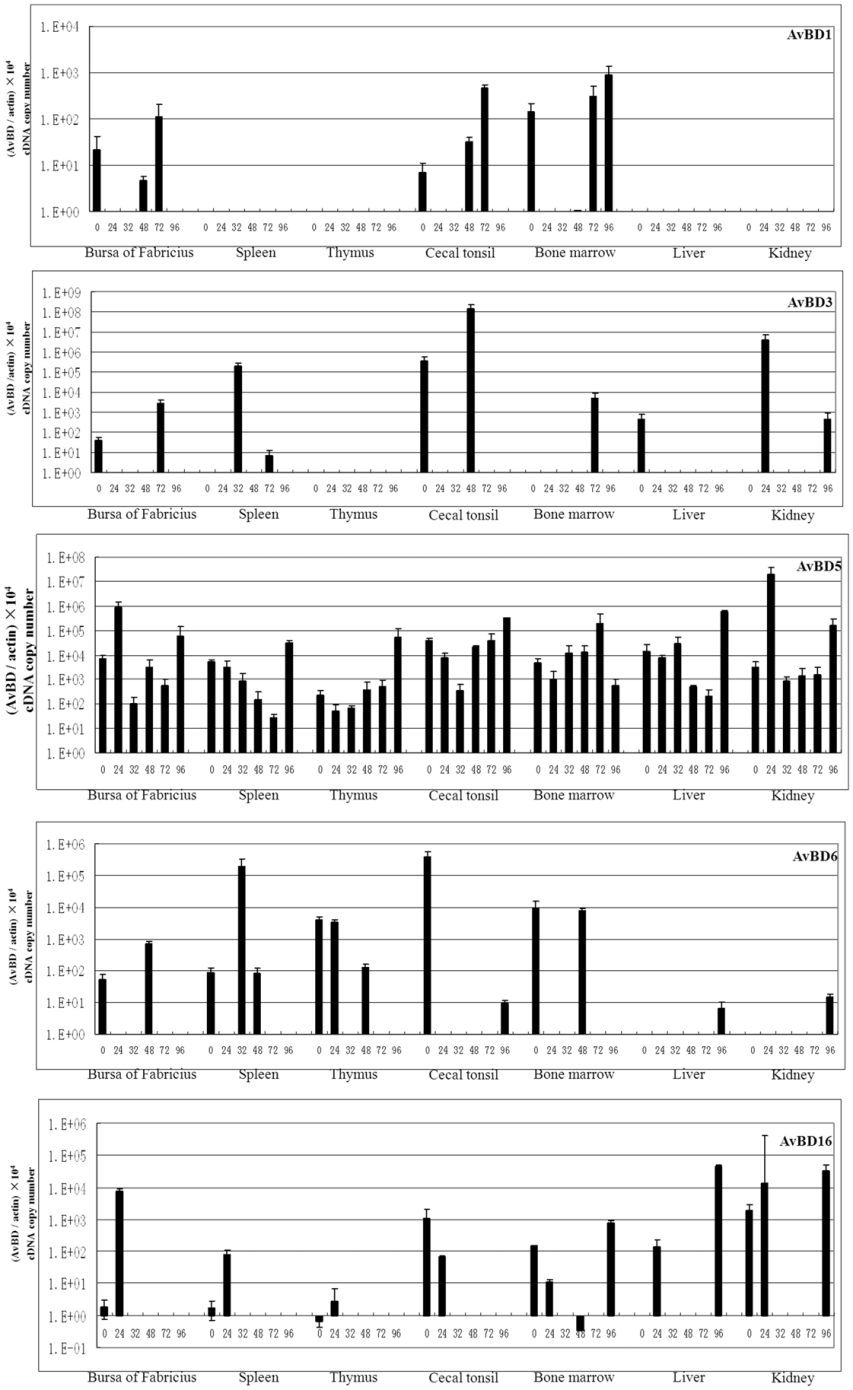
Induction of mRNA of Apl_avian β-defensins (Apl_AvBDs) in tissues from ducks infected with duck hepatitis virus. The cDNA copy number was measured by real time RT-PCR in tissues from five ducklings at 24, 32, 48, 72, and 96 h after infection, respectively. All assays were performed in three independent experiments, with three replicates per experiment, and each point is the mean ± SD. The data were analyzed using SAS [24].

In order to investigate whether the expression of AvBD mRNAs in tissues from ducks was constitutive or inducible in response to DHV infection, ducks were challenged by intramuscular inoculation with DHV-1. Birds were sacrificed at 0 h, and at 24, 32, 48, 72, and 96 h after infection, respectively. Seven tissues including the bursa of Fabricius, spleen, thymus, cecal tonsil, bone marrow, liver, and kidneys of each bird were collected and used to measure the level of mRNAs with the real-time RT-PCR method. The present results showed that, among the five novel Apl_AvBDs (Fig. 7), only Apl_AvBD5 was expressed widely in all tissues investigated from both the control and infected ducks. However, the mRNA expression of the other four novel Apl_AvBDs was highly variable. It was shown that Apl_AvBD1 was detected in the bursa of Fabricius, cecal tonsil, and bone marrow from the control ducks. Interestingly, the expression of Apl_AvBD1 was time dependent in these three tissues from the ducks after infection, and no obvious expression of the mRNA was detected in these three tissues from ducks at 24 and 32 h after infection. The levels of expression of the mRNA in the bursa of Fabricius and cecal tonsil at 72 h postinfection, and in bone marrow at 72 and 96 h postinfection, were significantly upregulated in response to infection (P<0.05 or P<0.01). The expression of Apl_AvBD3 was limited, and it was found only in the bursa of Fabricius, cecal tonsil, and liver in the control ducks. In contrast, the expression of AvBD was induced to a high level in spleen (32 and 72 h), bone marrow (72 h), and kidney (24 and 96 h) from ducks after infection (P<0.05 or P<0.01). The expression of Apl_AvBD5 was similar in most tissues investigated from both the control and infected ducks. It was significantly upregulated in the bursa of Fabricius (24 h), thymus (96 h), liver (96 h), and kidney (24 h) in response to infection (P<0.05 or P<0.01). With the exception of the liver and kidney, AvBD6 was detected in the other five tissues from the control. Significant upregulation of AvBD6 was observed only in the spleens of ducks after infection (at 32 h) (P<0.01). Significant induction was only observed for AvBD6 expression in both liver and kidney (at 96 h) from infected ducks. However, little expression of the mRNA was detected in any of the tissues investigated from the ducks at most time points after infection. Similar to the pattern of expression of AvBD6, AvBD16 was expressed at a low level in the tissues investigated, except for the liver from the control ducks. The level of expression of AvBD16 in both the bursa of Fabricius and the spleen was increased significantly at 24 h after infection (P<0.05 or P<0.01), and significant induction was observed in the liver (at 24 h and 96 h) in response to infection. However, little expression was observed in these tissues at most time points postinfection. In contrast to the pattern of expression of AvBD1, 3, 6 and 16 was expressed widely in all tissues from the ducks in both the control and infection groups. It was significantly upregulated in the bursa of Fabricius (at 72 h and 96 h) and spleen (at 24 h), thymus (at 24 h), cecal tonsil (at 24 h), bone marrow (at 96 h), and liver (at 32 h and 72 h) (P<0.05).

## Discussion

It is well documented that defensins display broad spectrum antimicrobial activity against Gram-positive and Gram-negative bacteria, as well as fungi and mycobacteria in vitro [15], [25]. In the current study we identified five novel AvBDs from ducks. Characterization, assessment of homology, and comparison of these genes with AvBDs from other avian species confirmed that they were Apl_AvBD1, 3, 5, 6, and 16. In the present study, GST-tagged peptides were used for analyzing antimicrobial activity, since it has been shown previously that the antimicrobial potency of these peptides is not to be significantly altered by the presence of tags including GST or His [8]–[14]. Similar to other β-defensins, the five novel Apl_AvBDs demonstrate broadspectrum antimicrobial activity against Gram-positive and Gram-negative bacteria. The mechanism of action of cationic antimicrobial peptides such as β-defensins is dependent mainly on electrostatic interactions between positive charges on the peptide sequence and the anionic surface of the outer membrane of Gram-negative bacteria [26], [27]. Comparison of the activities of the five Apl-AvBDs against Gram-negative bacteria showed that Apl_AvBD5, which is less cationic (+2.9) than Apl_AvBD1 (+8.89), Apl_AvBD3 (+5.06), Apl_AvBD6 (+7.89) or Apl_AvBD16 (+5.23), exhibits the weakest activity against most Gram-negative bacteria investigated. Interestingly, the N-terminal part is has a lower charge. This extremity, as proposed for the very similar ostrich defensins, could be important for antimicrobial activity against Gram-negative bacteria [28], [29]. However, there is no direct correlation between the net charge of the protein and its potency against Gram-negative bacteria, because AvBD5 (+2.9) and AvBD6 (+7.89) exhibit similar levels of antibacterial activity against *S. choleraesuis* and *P. multocida*. Moreover, the potencies of the five Apl_AvBDs against Gram-positive bacteria were comparable for most of the strains tested. Even if cationic charge is essential for antibacterial activity, no direct correlation between the net charge of the protein and its potency against Gram-positive bacteria can be established. Furthermore, in agreement with previous studies on other AvBDs and defensins from other species [12], [14], [30], sodium chloride (at a concentration of 150 mM) inhibited the antibacterial activity of the five novel AvBDs. In addition, none of the novel Apl_AvBDs showed hemolytic activity. These results suggest that these defensins are not toxic to animal cells at concentrations that are bactericidal.

In addition to the broad spectrum antibacterial potential, the defensin family of peptides have been shown previously to display antiviral activity towards influenza virus [31], adenovirus [32], and adeno-associated virus in vitro [33]. Among the AvBDs, Apl_AvBD4, 7, 12 were found to exhibit potent antiviral activity against DHV in our recent study [12]. Similarly, in this study we demonstrated that the five novel Apl_AvBDs inhibit replication of DHV in vitro. In addition to direct effects on the viral envelope, the antiviral activity of AMPs could also include blockage of viral entry through binding to components of the host, or through binding of viral glycoproteins. Furthermore, the cell-to-cell spread of viruses may be affected by AMPs, but so far no correlation between the structure or charge of the peptide and antiviral activity has been detected [30], [34]. The effect of the five novel Apl_AvBDs on these processes needs further investigation. In addition to enhancing our understanding of the role of defensins in innate host defense against viruses, these findings may point towards future therapeutic approaches for viral infections. Although we demonstrated that the five novel Apl_AvBDs may inhibit the replication of DHV, the mechanism by which they reduce the rate of replication remains to be determined.

Viruses have been shown to induce β-defensins in tissues following infection of animals in vivo [12], [35], [36]. Increased levels of β-defensins have also been observed in vitro following viral infection of human epithelial cells, and they have been associated clinically with virus infection [37], [38]. In lambs infected with parainfluenza virus type 3, the mRNA of sheep β-defensin-1 was increased in whole lung homogenates 17 days following infection [35]. In mice, murine β-defensin−1, −2, and −3 were induced in the lungs following infection with a mouse-adapted strain of influenza [39]. Similarly, it has been well documented that AvBDs are distributed extensively in various tissues in avian species [8]–[15]. Furthermore, the expression of most AvBD mRNAs in most organs is increased or induced following microbial infection, including those caused by viruses and bacteria in vivo [12], [14], [15]. Consistent with the results of previous studies, the mRNA expression of all five Apl_AvBDs in most tissues investigated was upregulated in response to DHV infection in the present study.

In summary, we identified and characterized, for the first time, five novel Apl_AvBDs (Apl_AvBD 1, 3, 5, 6, and 16) from ducks, and their antimicrobial activity against common pathogens, including bacteria and DHV, has been investigated. These peptides have antimicrobial activity against a broad range of bacteria and DHV, while their hemolytic activity is very low, even at high concentrations. Furthermore, the mRNA of the five novel Apl_AvBDs was upregulated in various immune tissues and in the livers of ducks following DHV infection. These findings provide evidence that the defensins activate the immune response to combat microbial infection.

## References

[1] Rodrigue DC, Tauxe RV, Rowe B (1990) International increase in Salmonella enteritidis: a new pandemic? Epidemiol Infect 105: 21–27. 2200698 10.1017/s0950268800047609 PMC2271793

[2] White DG, Zhao S, Sudler R, Ayers S, Fredman S, et al (2001) The isolation of antibiotic-resistant salmonella from retail groud meats. N Engl J Med 345: 1147–1154. 11642230 10.1056/NEJMoa010315

[3] Zasloff M (2002) Antimicrobial peptides of multicellular organisms. Nature 415: 389–395. 11807545 10.1038/415389a

[4] Brogden KA, Ackermann M, McCray Jr PB, Tack BF (2003) Antimicrobial peptides in animals and their role in host defences. Int J Antimicrob Agents 22: 465–478. 14602364 10.1016/s0924-8579(03)00180-8

[5] Ganz T (2005) Defensins and other antimicrobial peptides: a historical perspective and an update. Comb Chem High Throughput Screening 8: 209–217. 10.2174/1386207053764594 15892623

[6] Lynn DJ, Higgs R, Gaines S, Tierney J, James T, et al (2004) Bioinformatic discovery and initial characterisation of nine novel antimicrobial peptide genes in the chicken. Immunogenetics 56: 170–177. 15148642 10.1007/s00251-004-0675-0

[7] Lynn DJ, Higgs R, Lloyd AT, O’Farrell y C, Hervé-Grépinet V, et al (2007) Avian beta-defensin nomenclature: a community proposed update. Immunol Lett 110: 86–89. 17467809 10.1016/j.imlet.2007.03.007

[8] Ma DY, Liu SW, Han ZX, Li YJ, Shan AS (2008) Expression and characterization of recombinant gallinacin-9 and gallinacin-8 in Escherichia coli. Protein Expr Puri. 58: 284–291. 10.1016/j.pep.2007.11.017 18226919

[9] Ma DY, Liao WY, Wang RQ, Han ZX, Liu SW (2009) Two novel duck antibacterial peptides, avian beta-defensins 9 and 10, with antimicrobial activity. J Microbiol Biotechnol 19: 1447–1455. 19996700 10.4014/jmb.0904.4028

[10] Ma DY, Wang RQ, Liao WY, Han ZX, Liu SW (2009) Identification and characterization of a novel antibacterial peptide, avian beta-defensin 2 from ducks. J Microbiol 47: 610–618. 19851734 10.1007/s12275-009-0068-z

[11] Wang RQ, Ma DY, Lin LJ, Zhou CY, Han ZX, et al (2010) Identification and characterization of an avian β-defensin orthologue, avian β-defensin 9, from quails. Appl Microbiol Biotechnol 87: 1395–1405. 20396878 10.1007/s00253-010-2591-6

[12] Ma DY, Lin LJ, Zhang KX, Han ZX, Shao YH, et al (2011) Three novel Anas platyrhynchos avian β-defensins, upregulated by duck hepatitis virus, with antibacterial and antiviral activities. Molecular Immunology 49: 84–96. 21856003 10.1016/j.molimm.2011.07.019

[13] Ma DY, Lin LJ, Zhang KX, Han ZX, Shao YH, et al (2012) Discovery and characterization of Coturnix chinensis avian β-defensin 10, with broad antibacterial activity. Journal of peptide science 18: 224–232. 22389044 10.1002/psc.1437

[14] Ma DY, Zhou CY, Zhang MY, Han ZX, Shao YH, et al (2012) Functional analysis and induction of four novel goose (Anser cygnoides) avian β-defensins in response to salmonella enteritidis infection. Comp Immunol Microbiol Infect Dis 35: 197–207. 22285691 10.1016/j.cimid.2012.01.006

[15] van Dijk A, Veldhuizen EJ, Haagsman HP (2008) Avian defensins. Vet Immunol Immunopathol 124: 1–18. 18313763 10.1016/j.vetimm.2007.12.006 PMC7112556

[16] Olsen B, Munster VJ, Wallensten A, Waldenstrom J, Osterhaus AD, et al (2006) Global patterns of influenza A virus in wild birds. Science 312: 384–388. 16627734 10.1126/science.1122438

[17] Schägger H, von Jagow G (1987) Tricine-sodium dodecyl sulfate-polyacrylamide gel electrophoresis for the separation of proteins in the range from 1 to 100 kDa. Anal Biochem 166: 368–379. 2449095 10.1016/0003-2697(87)90587-2

[18] Bradford MM (1976) A rapid and sensitive method for the quantitation of microgram quantities of protein utilizing the principle of protein-dye binding. Anal Biochem 72: 248–254. 942051 10.1016/0003-2697(76)90527-3

[19] Available: http://www.oie.int/eng/publicat/en standards.htm.Accessed 2004 Jun 3.

[20] Shin SY, Park EJ, Yang ST, Jung HJ, Eom SH, et al (2001) Structure-activity analysis of SMAP-29, a sheep leukocytes-derived antimicrobial peptide. Biochem Biophys Res Commun 285: 1046–1051. 11467858 10.1006/bbrc.2001.5280

[21] Xiao YJ, Cai Y, Bommineni YR, Fernando SC, Prakash O, et al (2006) Identification and functional characterization of three chicken cathelicidins with potent antimicrobial activity. J Biol Chem 281: 2858–2867. 16326712 10.1074/jbc.M507180200

[22] Yu K, Park K, Kang SW, Shin SY, Hahm KS, et al (2002) Solution structure of a cathelicidin-derived antimicrobial peptide, CRAMP as determined by NMR spectroscopy. J Pept Res 60: 1–9. 12081622 10.1034/j.1399-3011.2002.01968.x

[23] Sadeyen JR, Trotereau J, Protais J, Beaumont C, Sellier N, et al (2006) Salmonella carrier-state in hens: study of host resistance by a gene expression approach. Microbes Infect 8: 1308–1314. 16702014 10.1016/j.micinf.2005.12.014

[24] SAS (1996) Institute. SAS User’s Guide: Statistics, SAS Institute Inc. Cary, NC.

[25] Lehrer RI, Ganz T (2002) Defensins of vertebrate animals. Curr. Opin. Immunol 14: 96–102. 10.1016/s0952-7915(01)00303-x 11790538

[26] Chan DI, Prenner EJ, Vogel HJ (2006) Tryptophan- and arginine-rich antimicrobial peptides: Structures and mechnisms of action. Biochemica Biophysica Acta - Biomembranes 1758: 1184–1202. 10.1016/j.bbamem.2006.04.006 16756942

[27] Powers JS, Hancock REW (2003) The relationship between peptide structure and antibacterial activity. Peptides 24: 1681–1691. 15019199 10.1016/j.peptides.2003.08.023

[28] Harwig SSL, Swiderek KM, Kokryakov VN, Tan L, Lee TD, et al (1994) Gallinacins: cysteine-rich antimicrobial peptides of chicken leukocytes. FEBS Lett 342: 281–285. 8150085 10.1016/0014-5793(94)80517-2

[29] Sugiarto H, Yu PL (2006) Identification of three novel ostricacins: an update on the phylogenetic perspective of β-defensins. Int J Antimicrob Agents 27: 229–235. 16459058 10.1016/j.ijantimicag.2005.10.013

[30] Veldhuizen EJ, Rijnders M, Claassen EA, van Dijk A, Haagsman HP (2008) Porcine beta-defensin 2 displays broad antimicrobial activity against pathogenic intestinal bacteria. Mol Immunol 45: 386–394. 17658606 10.1016/j.molimm.2007.06.001

[31] Ryan LK, Dai JJ, Yin ZW, Megjugorac N, Uhlhorn V, et al (2011) Modulation of human β-defensin-1 (hBD-1) in plasmacytoid dendritic cells (PDC), monocytes, and epithelial cells by influenza virus, Herpes simplex virus, and Sendai virus and its possible role in innate immunity. Journal of Leukocyte Biology. 90: 343–356. 10.1189/jlb.0209079 PMC3133436 21551252

[32] Bastian A, Schäfer H (2001) Human α-defensin 1 (HNP-1) inhibits adenoviral infection in vitro. Regul Pept 101: 157–161. 11495691 10.1016/s0167-0115(01)00282-8

[33] Virella-Lowell I, Poirier A, Chesnut KA, Brantly M, Flotte TR (2000) Inhibition of recombinant adeno-associated virus (rAAV) transduction by bronchial secretions from cystic fibrosis patients. Gene Therapy 7: 1783–1789. 11083501 10.1038/sj.gt.3301268

[34] Jenssen H, Hamill P, Hancock RE (2006) Peptide antimicrobial agents. Clin Microbiol Rev 19: 491–511. 16847082 10.1128/CMR.00056-05 PMC1539102

[35] Grubor B, Gallup JM, Meyerholz DK, Crouch EC, Evans RB, et al (2004) Enhanced surfactant protein and defensin mRNA levels and reduced viral replication during parainfluenza virus type 3 pneumonia in neonatal lambs. Clin Vaccine Immunol 11: 599–607. 10.1128/CDLI.11.3.599-607.2004 PMC404576 15138188

[36] Chong KT, Thangavel RR, Tang X (2008) Enhanced expression of murine β-defensins (MBD-1, -2,- 3, and -4) in upper and lower airway mucosa of influenza virus infected mice. Virology 380: 136–143. 18752820 10.1016/j.virol.2008.07.024

[37] Duits LA, Nibbering PH, van Strijen E, Vos JB, Mannesse-Lazeroms SPG, et al (2003) Rhinovirus increases human β-defensin-2 and -3 mRNA expression in cultured bronchial epithelial cells. FEMS Immunol Med Microbiol 38: 59–64. 12900056 10.1016/S0928-8244(03)00106-8

[38] Proud D, Sanders SP, Wiehler S (2004) Human rhinovirus infection induces airway epithelial cell production of human β-defensin 2 both in vitro and in vivo. J Immunol 172: 4637–4645. 15034083 10.4049/jimmunol.172.7.4637

[39] Chong KT, Xiang L, Wang X, Jun EL, Xi LF, et al (2006) High level expression of human epithelial β-defensins (hBD-1, 2 and 3) in papillomavirus induced lesions. Virol J 3: 75. 16961924 10.1186/1743-422X-3-75 PMC1579216

