# Correction: Identification, Expression and Activity Analyses of Five Novel Duck Beta-Defensins

**DOI:** 10.1371/annotation/8e3de33f-aaad-4c55-a21e-1677fe496f68

**Published:** 2013-04-30

**Authors:** Deying Ma, Kexin Zhang, Mingyue Zhang, Shengnan Xin, Xiaoli Liu, Zongxi Han, Yuhao Shao, Shengwang Liu

The following two publications by our group should have been cited in this PLOS ONE article, the authors apologize for this oversight:

Isolation, Characterization and Mechanism of Anti-viral Activity of Duck Avian Beta-defensin 16

Zhang K, Zhang M, Xin S, Han Z, Shao Y, Liu S and Ma D. Scientia Agricultura Sinica, 2012, 45(18): 3873-3882 (in Chinese)

Isolation, characterization, and determination on bioactivity of duck avian beta-defensin 5 Zhang K, Lin L, Han Z, Shao Y, Liu S and Ma D. Microbiology China, 2011, 38(11): 1688-1697.(in Chinese)

The sequences for Apl_AvBD16 and Apl_AvBD5 used for the study reported in PLOS ONE are identical with respect to AvBDs from the two reports in Chinese above, however, all the experiments for AvBD 16 and AvBD 5 were repeated to generate new datasets to be reported in the PLOS ONE article. Furthermore, the data for Apl_AvBD1, 3, and 6 reported in the PLOS ONE article were not included in the studies of the two Chinese publications above.

The claim in the PLOS ONE article indicating that this reports the characterization of five novel defensins is inaccurate given that beta-defensin 16 and beta-defensin 5 had been already reported in the two previous publications above. 

